# pH-Driven Selective Adsorption of Multi-Dyes Solutions by Loofah Sponge and Polyaniline-Modified Loofah Sponge

**DOI:** 10.3390/polym14224897

**Published:** 2022-11-13

**Authors:** Melissa G. Galloni, Veronica Bortolotto, Ermelinda Falletta, Claudia L. Bianchi

**Affiliations:** 1 Department of Chemistry, University of Milan, Via Golgi 19, 20133 Milan, Italy; 2 Consorzio Interuniversitario Nazionale per la Scienza e Tecnologia dei Materiali (INSTM), Via Giusti 9, 50121 Florence, Italy

**Keywords:** dyes, polyaniline, loofah, adsorbents, water remediation, easy recovery

## Abstract

In the last decades, sorbent materials characterized by low selectivity have been developed for the removal of pollutants (in particular dyes) from wastewater. However, following the circular economy perspective, the possibility to selectively adsorb and desorb dyes molecules today represents an unavoidable challenge deserving to be faced. Herein, we propose a sequential treatment based on the use of PANI-modified loofah (P-LS) and loofah sponge (LS) to selectively adsorb cationic (rhodamine, RHB, and methylene blue, MB) and anionic (methyl orange, MO) dyes mixed in aqueous solution by tuning the adsorption pH (100% MO removal by P-LS and 100% and 70% abatement of MB and RHB, respectively, by LS). The system maintained high sorption activity for five consecutive cycles. A simple and effective regeneration procedure for the spent adsorbents permits the recovery of the initial sorption capability of the materials (81% for MO, *ca.* 85% for both RHB and MB, respectively) and, at the same time, the selective release of most of the adsorbed cationic dyes (50% of the adsorbed MB and 50% of the adsorbed RHB), although the procedure failed regarding the release of the anionic component. This approach paved the way to overcome the traditional procedure based on an indiscriminate removal/degradation of pollutants, making the industrial wastewater a potential source of useful chemicals.

## 1. Introduction

Dyes find large applications in textile, pharmaceutical, food, paint, chemical, cosmetic, printing, paper, gasoline, lubricants, oils, soaps, detergents, etc. [1]. However, their release in water matrices represents a global worry, owing to their wide use in several industries.

Unfortunately, even small amounts of these pollutants can drastically affect the quality of water bodies due to their influence on light penetration reduction. In this way, they disturb the photosynthetic activities of aquatic life, inducing dramatic consequences on the aquatic flora [2,3].

Moreover, because of their carcinogenic and mutagenic effects, it has been demonstrated that dyes have long-term adverse effects on humans and terrestrial animals [4]. For all these reasons, the production of large volumes of dye-loaded wastewater is a severe concern, in particular in countries that still do not have restrictive environmental regulations. It is important to highlight that removing dyes from wastewater effluents is a complex process that conventional methods, such as physical, chemical, physico-chemical, and biological treatments, hardly perform efficiently [5,6,7].

Over the years, several technologies have been proposed for dyes’ abatement, such as adsorption, coagulation, filtration, ion exchange, precipitation, electrodialysis, membrane separation, oxidation, etc. [8,9,10,11,12,13].

Among them, adsorption on solids achieves very fast and cost-effective removal of water pollutants [12,13,14,15]. Still, these processes simply transfer the contaminants from water to another phase (usually a solid substrate) [16], which then needs additional treatment and/or disposal, leaving the user with a waste to manage. Activated carbons represent the most used adsorbents thanks to their low selectivity, high efficiency, and stability [17,18,19,20,21,22], even if the necessity of high temperatures or toxic organic solvents for their regeneration makes them poor in terms of sustainability and enhances the process cost.

Moreover, although the most common way of applying the effluent treatment on a large scale is based on the use of fixed beds, the possible use of adsorbents in the form of dispersed powders (slurry configuration) poses severe problems for large-scale industrial applications, where the recovery of suspended particles is an issue [23,24,25].

Although, so far, materials characterized by low selectivity have been studied for the removal of pollutants, today, new emerging investigations are based on the selective recovery of products rather than on their indiscriminate removal/degradation. In fact, from a circular economy perspective, industrial wastewater should be considered as a potential source of valuable chemicals to save raw materials. In this context, a selective recovery of dyes from effluents derived by textile industries should represent an important goal, albeit the presence of multi-component dyes in complex matrices could be a strong limitation for traditional adsorbents. However, the scientific community should seriously address this topic by developing easily recoverable, highly selective adsorbents.

Recently, conducting organic polymers (COPs) have emerged as interesting alternatives to traditional adsorbents thanks to their unique chemical and physical properties. Among them, polyaniline (PANI) is unique for its interesting multifunctionality, easy synthesis, high chemical and environmental stability, porosity, and high surface area. For this reason, it has been extensively investigated as an active material for sampling [26] and solid phase extraction [27,28,29] and as a sorbent for water pollutants removal [30,31,32,33,34].

However, over the years, polysaccharides of vegetable origin, such as coconut and rice husks, tobacco dust, palm fruit fibers, and many others [35,36,37,38], have been investigated as an alternative to traditional and/or synthetic sorbents for dyes removal. They are promising biopolymers thanks to their high availability in nature, low cost, biodegradability, renewable characteristics, high stability, and ease of modification.

Recently, loofah sponge and its composites have been employed as potential adsorbents for removing dyes from aqueous solutions, even if in different experimental conditions [39,40,41,42]. In this regard, Altinişik and coworkers demonstrated that loofah cylindrica can be successfully used as an efficient adsorbent for removing malachite green from aqueous solution at pH values of 3–5, reaching the sorption capacity of 29.4 mg·g^−1^ [39]. Similarly, Mashkoor and Nasar proposed chemically modified loofah aegyptica for the adsorption of malachite green, raising the material performance up to 78.79 mg·g^−1^ [40].

Loofah composites were also fabricated to enhance the performance of the bare biomaterials. In more detail, the attempt of Qiang et al. to cover the loofah sponge with Ca-alginate led to the removal of methylene blue at pH 6.50, guaranteeing the sorption capacity of about 180 mg·g^−1^ [41]. On the other hand, the modification of the loofah surface with zinc nanoparticles did not strongly affect its sorption capability towards trypan blue, passing from 45.3 mg·g^−1^ to 47.3 mg·g^−1^ [42].

Herein, we propose a two-step, pH-driven, fast, reversible and selective adsorption process for removing dyes from aqueous mixtures by combining the natural loofah sponge (LS) and PANI-modified loofah sponge (P-LS). A thorough batch of adsorption experiments was conducted for the selective removal of rhodamine B, methylene blue, and methyl orange in single-dye solutions and their mixtures. Furthermore, kinetic investigations demonstrated that the pseudo-second-order kinetic model represents the adsorption kinetics of all the three dyes. Moreover, the separation of multi-dye mixtures (methyl orange, rhodamine, and methylene blue) was additionally tested in consecutive cycles. Finally, the possibility to quickly regenerate the adsorbents systems for their reuse and selectively recover the adsorbed dyes was explored. Results demonstrated that a simple post-treatment of the materials permits, on the one hand, the recovery of much of the initial adsorbing capacity and, on the other hand, the selective recovery of 50% of both the adsorbed MB and RHB, respectively, unlike MO, which is completely held back.

## 2. Materials and Methods

### 2.1. Materials

All chemicals of analytical grade were purchased from Sigma-Aldrich (Merck & Co, St. Louis, MO, USA), whereas loofah sponge was bought by Happyfarma. Single-dye solutions were prepared by dissolution of 10 mg of each dye (rhodamine B, RHB; methyl orange, MO; methylene blue, MB) in 1 L of ultrapure water (dye concentration: 10 mg·L^−1^). The dyes’ mixture was prepared by dissolving 10 mg of each dye in 1 L of ultrapure water (total concentration of dyes: 30 mg·L^−1^; concentration of each dye: 10 mg·L^−1^). For HPLC/UV analyses, HPLC-grade acetonitrile and water were purchased from Carlo Erba reagents.

### 2.2. Sample Preparation

**Scheme 1:**
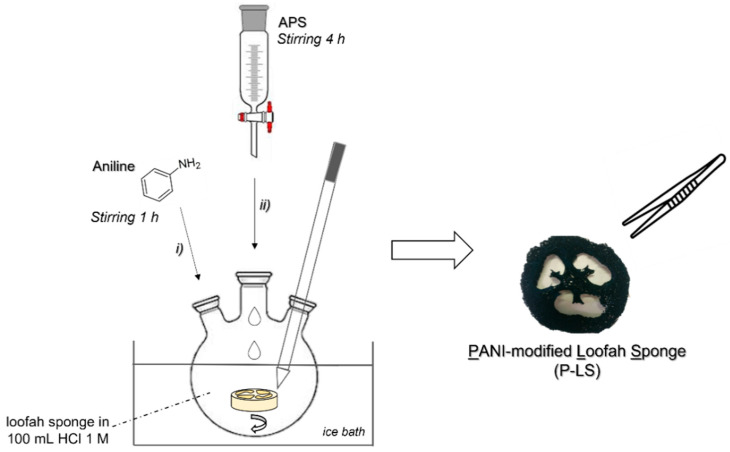
Representation of a typical P-LS preparation.

LS was cut to obtain slices of about 0.60 g (Scheme 1). Before use, each sample was washed in ethanol under stirring (250 rpm) for 1 h to remove impurities and then dried at room temperature.

For the preparation of P-LS, an in situ polymerization method was used. In a typical experiment, 1 mL aniline (11 mmol) was dissolved in 90 mL HCl 1 M (HCl: aniline = 10 molar ratio) and stirred for 1 h in an ice bath in the presence of the loofah sponge. Then, 100 mL ammonium persulphate (APS) 0.1 M prepared in 1 M HCl was added dropwise to the aniline solution and stirred for 4 h (APS: aniline = 1.5 molar ratio). The as-obtained P-LS showed a dark green colour. It was easily recovered by tweezers, dried at room temperature, washed several times with deionized water, and then air-dried again.

Scheme 1 reports the P-LS preparation method described above.

**Figure 1:**
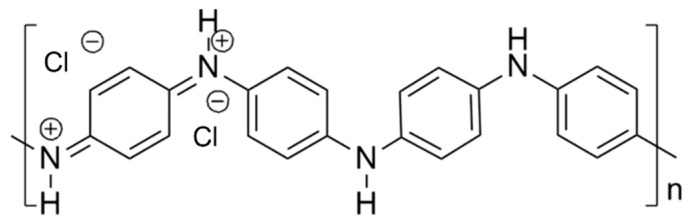
Structure of PANI in form of emeraldine salt (for the tetrameric unit n = 1).

The percentage of PANI grown on LS was computed on the basis of the elemental (CHN) analysis by Equation (1).

(1) $$wt.{\%}_{P/P-LS}=\frac{\frac{\%N_{CHN}}{MW_{N}}}{4}\cdot MW_{PANI}$$

where *N% _CHN_* is the percentage of nitrogen obtained by the CHN analyses, 4 indicates the number of N atoms in the tetrameric unit for PANI as emeraldine salt (Figure 1), and MW_PANI_ is the molecular weight of the tetrameric unit.

The percentage of PANI on LS corresponded to *ca.* 6.5 wt.%.

### 2.3. Characterization

Infrared spectra of both LS and P-LS were recorded using a spectrophotometer (Thermo Nicolet, iS10, Thermo Fisher, Waltham, MA, US) under the attenuated total reflectance (ATR) method in the 4000–600 cm^−1^ interval. The morphology was studied by field emission scanning electron microscopy using a SEM Leo 438 VP (Zeiss, Overcoached, Germany) without any pre-treatment of the samples.

CHN analyses were carried out by a Perkin Elmer CHN 2004 (Waltham, MA, USA). Before the analyses, the samples were finely chopped.

Specific surface area values were determined by N_2_ adsorption/desorption isotherms −196 °C using an automatic analyser of surface area (Coulter SA3100 instrument, Beckman Life Sciences, Los Angeles, CA, USA). Before the analysis, the dried sample (*ca.* 0.50 g) was outgassed at 150 °C for 4 h under vacuum with the final aim to remove water and other volatile organic compounds adsorbed on the surface. Specific surface area values were calculated by Brunauer–Emmet–Teller (BET) equation (2-parameters, 0.05 < p/p^0^ < 0.3), considering a cross-sectional area of *ca.* 16.2 Ǻ/molecule_N2_.

The point of zero charge (PZC) of LS and P-LS was determined according to the following procedure, previously reported in the literature [43]. In a typical experiment, *ca.* 50 mg of each sample was weighed and introduced in NaNO_3_ solutions (20 mL, 0.1 M) under stirring. Initial pH values (pH_initial_) of NaNO_3_ solutions were adjusted in the 4.00–10.00 interval by properly adding 0.1 M HNO_3_ or NaOH. Suspensions were maintained under stirring (250 rpm) for 24 h, and, successively, the final pH values (pH_final_) were measured after the samples’ removal. By plotting the difference between the pH_final_ and pH_initial_ (∆pH) along with the pH_initial_, pH_pzc_ was determined as the intersection of the resulting line at which ∆pH = 0.

### 2.4. Adsorption Tests

#### 2.4.1. One-Step Adsorption Test for Dyes Removal

LS and P-LS were firstly tested as adsorbents in the removal of a single dye from the ultrapure water matrix at spontaneous pH and at pH 3 and 8. Each material was immersed in 100 mL of a single dye solution having a concentration of 10 ppm. The solution was maintained under stirring at room temperature for 210 min, withdrawing aliquots after every 30 min. All the aliquots were analysed by UV-vis spectroscopy (T60 UV-visible Spectrophotometer PRIXMA (PG instruments, Lutterworth, United Kingdom) at the following wavelengths: 508 nm for MO, 554 nm for RHB and 678 nm for MB.

The procedure was repeated using a mixture of the three dyes (10 ppm each, for a total concentration of 30 ppm) at spontaneous pH.

#### 2.4.2. Two-Step pH-Triggered Adsorption of Dyes in a Mixture

P-LS was immersed in 100 mL of dyes mixture solution having a concentration of 30 ppm (10 ppm for each dye) for 60 min under stirring (250 rpm), withdrawing aliquots after 15, 30, and 60 min. After that, the P-LS was removed; then, 0.1 M HCl was dropwise added to the solution until pH 3. Then, LS was immersed in the solution and stirred (250 rpm) for 150 min, withdrawing aliquots every 15 min for the first half hour and then every 30 min. At the end, LS was removed from the solution. Both P-LS and LS were washed with water and dried at room temperature.

All the aliquots were analysed *via* HPLC with 50% CH_3_CN, 50% H_2_O, 0.1% HCOOH as eluent and 1 mL·min^−1^ as flowrate. Each analysis lasted 11 min, and the selected wavelengths were 508 nm for MO, 554 nm for RhB, and 678 nm for MB. The HPLC instrument (Agilent 1100 Series, Agilent, Santa Clara, CA, USA) was equipped with a C18 Supelco column (25 cm, 4 mm, 5 m), a 20_L autosampler, and a UV detector.

#### 2.4.3. Recycles, Desorption Tests, and Materials’ Regeneration

The adsorption test, described in the Section 2.4.2., was repeated for another four cycles without any other post-treatment to evaluate the reusability of P-LS and LS.

Finally, to desorb the dyes from each adsorbent, both P-LS and LS were immersed at first in a pH 8 NH_3_ solution for 30 h and then washed in a 0.1 M HCl solution for 72 h. The materials were washed with water, then dried at room temperature, and reused.

All the solutions, containing the desorbed dyes, were analysed *via* HPLC as described above.

## 3. Results and Discussion

### 3.1. Materials Characterization

Both LS and P-LS were characterized by different techniques.

**Figure 2:**
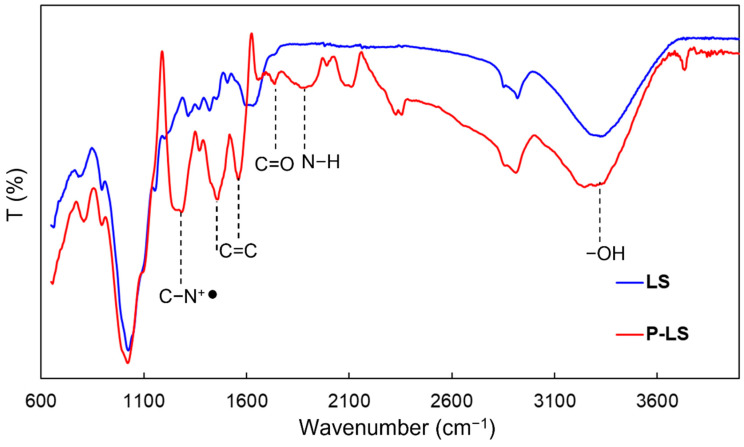
ATR FT−IR spectra of LS and P−LS.

Chemical structures of pristine loofah sponge (LS) and PANI-modified loofah sponge (P-LS) were characterized by ATR FT-IR spectroscopy, as reported in Figure 2.

According to the literature [19,33,40], the broadband at about 3293 cm^−1^ can be attributed to the -OH stretching vibration in cellulose, hemicellulose, and monosaccharide molecules, whereas the band at 2893 cm^−1^ is assigned to the -CH stretching of -CH and -CH_2_ groups in cellulose and hemicellulose components. The signal at 1705 cm^−1^ is related to the C=O stretching vibration in lignin and hemicellulose. The C=C aromatic conjugated bond tensile vibrations, characteristic of lignin, are responsible for the band at 1616 cm^−1^ and the benzene ring stretching (lignin) is responsible for the band at 1471 cm^−1^. Eventually, the band at 1016 cm^−1^ could be assigned to the C–O bonds in cellulose and hemicellulose.

After PANI modification, the broadband of the -OH stretching vibration of LS was shifted to 3236 cm^−1^, indicating that an interaction between PANI’s amine groups and LS’s OH groups occurred. Moreover, new signals, typical of the conducting polymer in its emeraldine form (half-oxidized, half-protonated), were detected. In particular, the band at 1435 cm^−1^ is related to C=C stretching vibrations of benzene rings of the polymer, whereas that at 1545 cm^−1^ to those of quinone rings [9,30,31,32,33,44], and the signal at 1250 cm^−1^ was assigned to the C–N^+^• stretching vibration modes [45].

The other peaks in the 2000–1800 cm^−1^ range can be associated with C–H vibrations in the polymer structure, whereas those at 1722 cm^−1^ and at 1647 cm^−1^ are associated with the presence of C=O and N-H vibrations.

**Figure 3:**
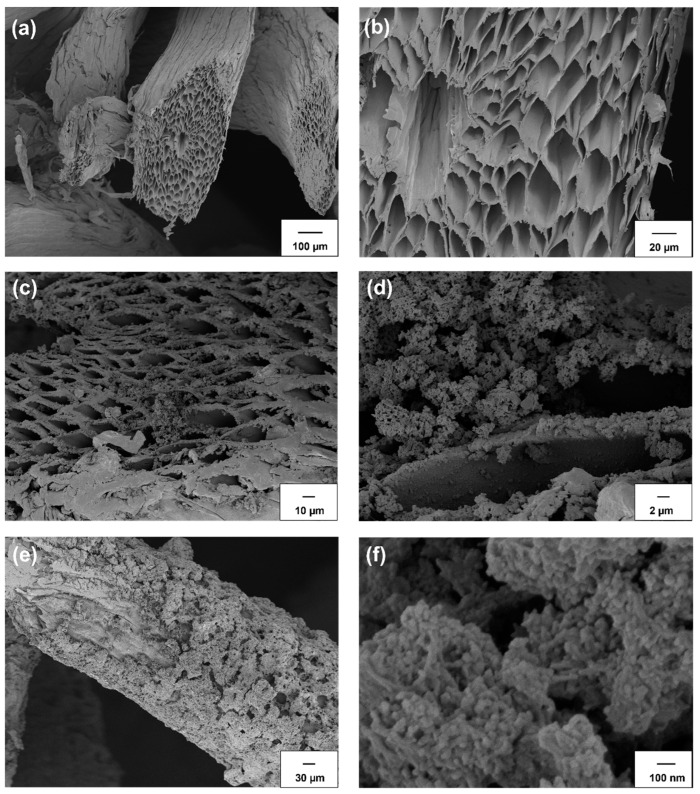
SEM images of LS (**a**,**b**) and P-LS (**c**–**f**).

The morphology of the materials was investigated by SEM. The collected micrographs (Figure 3) reveal the surface and cross-sectional characteristics of both LS and P-LS.

As expected, LS is characterized by a hollow three-dimensional network structure guaranteeing excellent structural stability and high porosity (Figure 3a,b). Figure 3 c–f shows the PANI discontinuous growth on the surface of LS in form of mixed rod-like and globular-like aggregates, increasing the roughness of the LS surface. In more detail, the rods were, on average, 150 nm long and 30 nm wide. In addition, N_2_ adsorption/desorption isotherms at −196 °C were also collected for both LS and P-LS samples. Figure S1 reports the obtained isotherms in the case of the LS sample. In general, according to the literature [46], a very low value of specific surface area for LS was found (*ca.* 0.15 m^2^·g^−1^). The collected isotherms (Figure S1) are typical of non-porous materials [47] and no hysteresis was detected. When PANI was introduced on LS, no variation in the surface area value was detected due to the very low amount of the polymer on the surface of the sponge (*ca.* 6.5 wt.%). Therefore, if on one hand these results justify the low adsorption capacity of the materials compared to other systems, on the other hand, they demonstrate the role of the surface interaction on the selective removal of the pollutants in solution.

**Figure 4:**
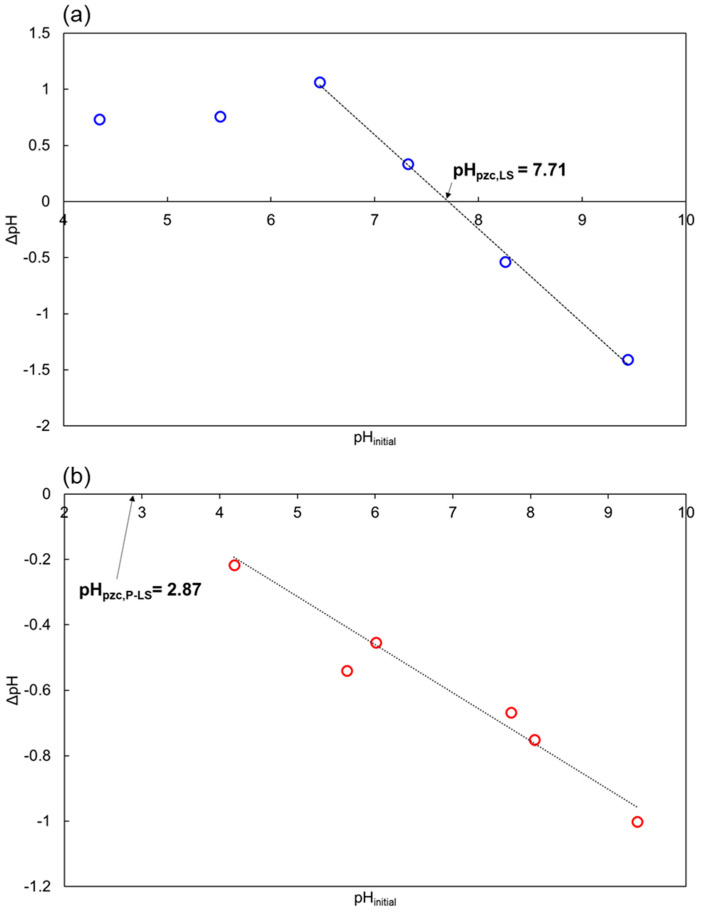
Point of zero charge of LS (**a**) and P−LS (**b**).

The determination of the point of zero charge is of paramount importance in surface characterization because it determines how easily a substrate can potentially adsorb harmful ions. Figure 4 displays the variations of pH solution caused by the presence of both LS and P-LS as a function of the initial pH solution (ΔpH vs. pH_initial_) and the corresponding point of zero charge (PZC). The latter describes the condition when the electrical charge density on a surface is zero (isoelectric point, PI).

According to the literature [48,49], the PZC of LS is 7.71. This demonstrates that, at the pH values lower than pI (that is, the pH at PZC), the LS surface is positively charged thanks to the presence of carboxyl and hydroxyl groups; whereas, at pH values greater than pI, negative charges prevail on the surface.

Concerning P-LS, the PZC value is shifted at lower pH values, 2.87, as previously observed for PANI-based materials [33]. For this system, at pH below 2.87, nitrogen atoms, in particular imine groups, are easily protonated [48], positively charging the surface of PANI. In contrast, at pH values higher than the pH_PZC_, P-LS is negatively charged. Based on these results, the performances of both the sorbents (LS and P-LS) at different pH values can be predicted, also as a function of the pollutants’ charge.

### 3.2. Adsorption Studies

Both adsorbents, LS and P-LS, were tested in the removal of different types of dyes (anionic and cationic).

**Figure 5:**
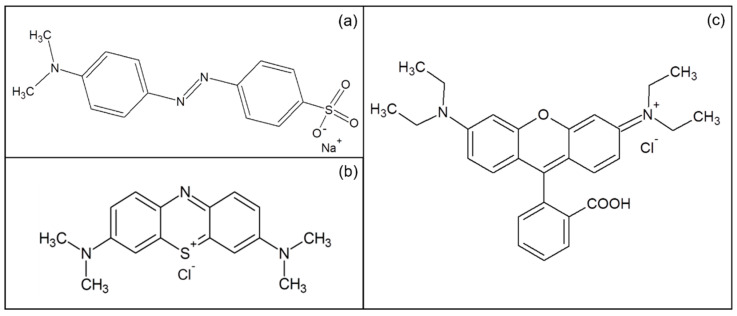
Molecular structure of (**a**) MO, (**b**) MB, and (**c**) RHB.

Methyl orange (MO) was selected as a unique model molecule for anionic dyes, whereas, concerning cationic dyes, two different molecules were selected: rhodamine (RHB) as the model molecule for xanthenes, and methylene blue (MB) as the model molecule for thiazines (Figure 5).

For each material, the best adsorption conditions were achieved by properly setting the initial pH of the solution, as described below.

#### 3.2.1. Effect of Initial pH on Adsorption Properties

The pH solution is a crucial controlling parameter in the adsorption processes, acting on the surface charges of the sorbent, as well as on the ionization status of the dye [50,51,52]. In addition, as reported above, on the basis of the PZC results, it should be possible to predict the sorbents’ performances. In general, at high pH values, the adsorbents’ surface becomes more negatively charged, promoting the adsorption of positively charged adsorbates. In contrast, at low pH values, the adsorbents’ surface becomes more positive, and the adsorption of negatively charged adsorbates should be more efficient.

**Figure 6:**
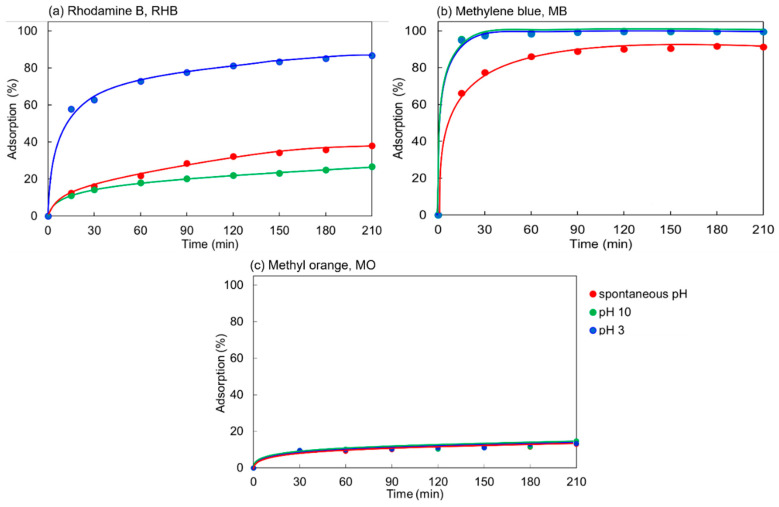
Effect of initial pH on the adsorption properties of LS towards (**a**) RHB, (**b**) MB, and (**c**) MO.

Figure 6 shows the effect of the initial pH value of the solution on the sorption properties of LS towards the selected dyes.

First of all, the adsorption tests were carried out at spontaneous pH to avoid, if possible, the addition of chemicals into the solution. Then, adsorption tests were repeated by properly modifying the initial pH to investigate the materials’ properties and, when necessary, enhance the adsorption capability of the sorbent.

For all the three dyes, the pH value of their aqueous solution was almost neutral (pH 6.8–7.5).

Concerning RHB adsorption, the effect of the initial pH of the solution is a complex process. In this regard, Wang and Zhu demonstrated that the pH exerted little effect on RHB adsorption on different solids owing to the presence of different functional groups on the molecule [53]. However, this is not true when LS is used as the adsorbent. On the basis of our knowledge, this is the first investigation into the adsorption activity of LS towards this type of cationic dye. RHB can exhibit different molecular forms in different pH conditions: the protonated monomeric form below pH = 3.5 and the zwitterionic form above this pH value [53,54]. According to the PZC measured for LS, at spontaneous pH, the number of both acidic and basic functional groups on the LS surface can be considered approximately equal. In contrast, RHB is present in solution in zwitterionic form. Both these aspects limit the effective interactions between the dye and the material surface (Figure 6a, red line). Similar results were obtained in alkaline conditions (Figure 6a, green line), since, even though it was at pH 10, negative charges prevailed on the surface of LS; the types of interactions occurring between RHB and LS are not relevant.

On the other hand, the sorption capability of LS drastically rose up when the initial pH value was lowered to 3 (Figure 4a, blue line). In these conditions, positive charges prevail on the surface of the sorbent, permitting hydrogen-bond interactions with RHB molecules and leading to the removal of RHB from the matrix.

If compared to RHB, the molecular structure of MB is quite independent of the pH solution, maintaining the same cationic characteristics in any conditions. As a consequence, the change in pH was expected to have only an effect on LS.

The sorption activity of LS towards MB is already high at spontaneous pH, achieving 91% of MB removal, and it approaches 100% when the surface of the absorbent takes on net charges (both positive and negative).

Unlike the results obtained for cationic dyes, LS seems to have no affinity towards anionic ones (Figure 6c).

**Figure 7:**
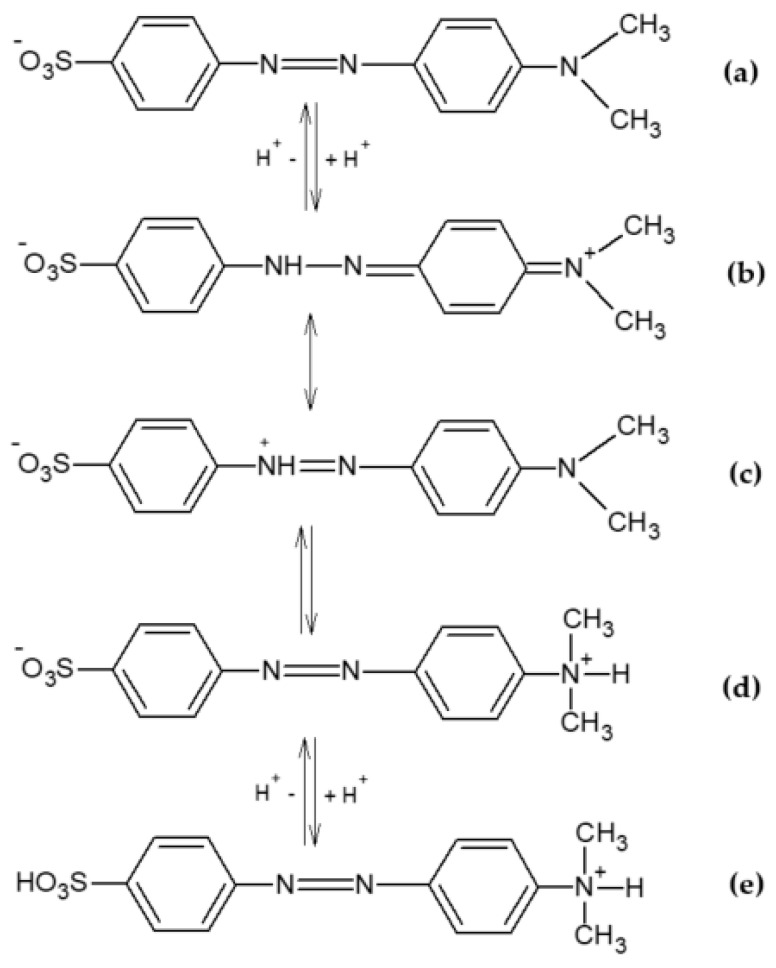
Different MO structures: (**a**) alkaline, (**b**–**d**) zwitterionic and its tautmer, and (**e**) acidic.

The scientific literature lacks investigations in this regard. In addition, the chemistry of MO at different pH conditions is complex, as confirmed by Del Nero et al. [55]. In fact, in alkaline conditions, MO exists as an anion (Figure 7a), and the N=N double bond represents a well-defined barrier for the *cis-trans* isomerization, conferring a certain rigidity to the molecular structure. The latter, along with the weak basic character of the alkaline anion, precludes any efficient interaction with the sorbent.

Under progressive protonation, the monoprotonated form of MO prevails in the solution. It can exist as a resonance hybrid between its quinine diimine and azonium structures (Figure 7b,c, respectively). For these zwitterionic structures, the double-bond character of the NN bond is lessened, reducing the rigidity of the structure around this bond.

The zwitterionic structures presenting charge separation are the most stable forms even at acidic pH (pH > pKa = 3.4), limiting the capacity of the charges on the absorbent surface to interact effectively with the dye molecules. Similarly, at very low pH values (pH < pKa = 3.4), the cationic form of MO prevails, promoting the repulsion phenomena with the positive charges on the sorbent.

These results demonstrate that, for the removal of some pollutants, such as dyes, the molecule/sorbent interaction depends on several factors, strictly related to the dye’s structure, pH of the solution, surface charges of the adsorbent and its capability to act by several interactions (e.g., hydrogen bonds), etc. As a consequence, the evaluation of PZC alone is not sufficient to predict the behavior and adsorbing properties of a solid.

However, the results showed that if, on the one hand, LS is a very promising candidate for cationic dyes removal and its selectivity can be easily tuned by working on the pH value of the solution, on the other hand, its sorption capability towards anionic dyes is poor.

In this context, with the aim to enhance the sorption properties of LS towards MO and, more in general, towards anionic dyes, the pristine sponge was properly modified, as described above, by a porous coating of PANI in the form of emeraldine salt. In fact, the scientific community has extensively demonstrated that PANI-based materials are good candidates for the removal of different types of pollutants in different matrices, including anionic dyes [30,32,55,56].

**Figure 8:**
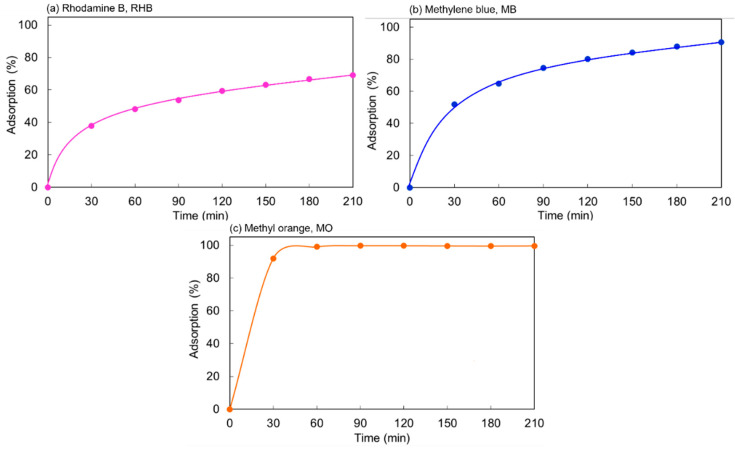
Adsorption capability of P-LS towards (**a**) RHB, (**b**) MB and MO (**c**).

Figure 8 displays the adsoption properties of P-LS towards the removal of the same dyes at spontaneous pH.

**Figure 9:**
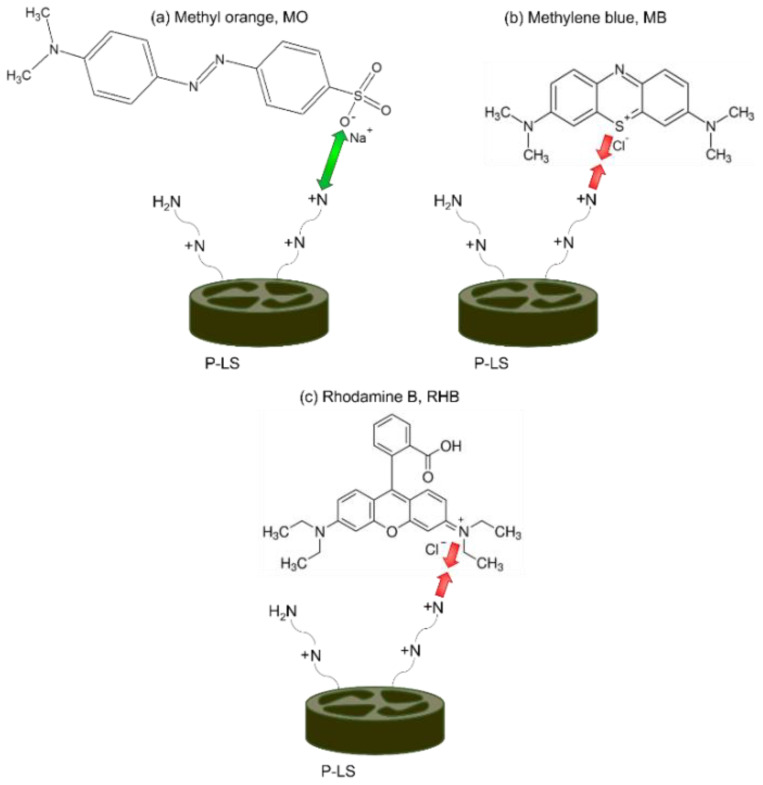
P−LS interaction with methyl orange (**a**), methylene blue (**b**), and rhodamine−B (**c**).

As expected, P-LS shows a very high affinity towards anionic dyes thanks to the interaction between the cationic protonated imines sites of PANI and the negative charge of the sulfonic group of MO (Figure 9a).

In contrast, on the basis of the scientific literature [33], emeraldine salt should exhibit poor adsorption capability towards cationic species because of the repulsions between the positively charged imine groups of PANI and the cationic pollutants in solution (Figure 9b,c).

However, the activity of P-LS to remove both MB and RHB is surprisingly impressive (*ca.* 80% for both dyes).

The high sorption activity of the composite towards both the anionic and cationic dyes can be explained by the not-homogeneous distribution of the polymer on the sponge’s surface, as confirmed by the SEM results (Figure 3c–f), displaying PANI-rich zones that alternate with others where the bare loofah prevails. This permits the combination of the properties of the conducting polymer with those of the pristine loofah, thus resulting in an enhanced unselective adsorption ability of the final composite. In addition, the PANI–loofah interaction plays a synergistic role in the RHB abatement.

#### 3.2.2. Adsorption Kinetics

**Figure 10:**
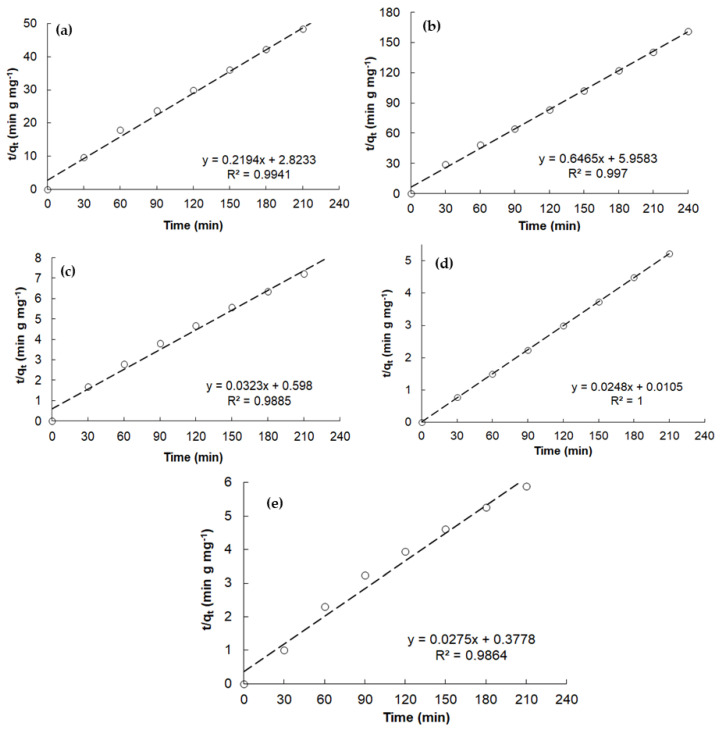
Pseudo−second-order kinetic model for: (**a**) RHB adsoption by LS at pH 3, (**b**) MB adsorption by LS at spontaneous pH, (**c**) RHB adsoption by P−LS at spontaneous pH, (**d**) MO adsorption by P−LS at spontaneous pH, (**e**) and MB adsorption by P−LS at spontaneous pH.

The experimental data reported above were applied to carry out the kinetic studies of RHB, MB, and MO dyes adsorption by both LS and P-LS by four kinetic models: pseudo-first-order, pseudo-second-order, Elovich, and intraparticle diffusion. Figure 10 and Figure S2 depict the results obtained for each kinetic model.

The pseudo-first-order model is also known as the Largergren model: it is based on the proportionality between the number of sites occupied by adsorption and the unoccupied sites. By this model, it is defined that the adsorbate and the surface of the adsorbent bind only to a single active site and the type of interactions are of a physical nature.

The pseudo-second-order kinetic model assumes that the adsorption follows the square of the difference between the number of adsorption sites available and the number of occupied sites and that the adsorbate can bind to two active sites with different binding energies. The Elovich model is similar to the latter and assumes that the surface of the adsorbent is heterogeneous and considers adsorption based on chemisorption phenomena. Finally, the intraparticle diffusion model, known also as the Morris–Weber model, mathematically demonstrates that the adsorption kinetics process is not influenced by the diffusion phenomenon of adsorbate in the adsorbent [57].

Kinetic investigations demonstrated that, in all cases, dye adsorptions follow pseudo-second-order kinetics (Equation (2)):

(2) $$\frac{t}{q_{t}}=\frac{1}{{\left(K_{2}q_{e}\right)}^{2}}+\frac{t}{q_{e}}$$

where q_e_ and q_t_ represent the amount of dye absorbed at equilibrium and for each time analyzed and K_2_ are obtained from the inverse values of the y-intercept and slope, respectively, of the plot of t/qt versus t, presented in Figure 10.

In addition, the maximum adsorption capacity of LS and P-LS towards the investigated dyes and a comparison with the scientific literature are reported in Table 1.

As reported in Table 1, the adsorption capacity of loofah-based adsorbents is affected by several factors (i.e., type of modification, pH of the solution, dye’s concentration, temperature, etc.), and, as a consequence, a direct comparison among the results can not be carried out.

However, it is evident that the PANI modification leads to an outstanding increase in the maximum adsorption capacity of the natural sponge, permitting LS to achieve results in general in line with those reported in the literature and, for some dyes (MO), more promising.

#### 3.2.3. Investigation of the Adsorption Properties of LS and P-LS towards Dyes in a Mixture

**Figure 11:**
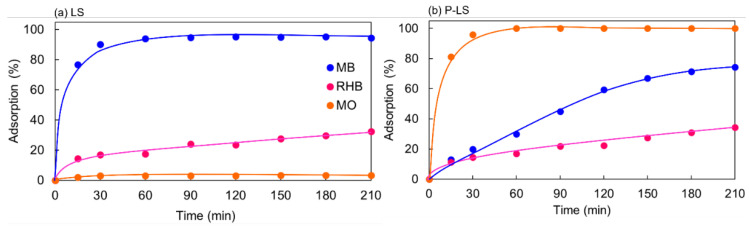
Adsorption capability of both LS (**a**) and P-LS (**b**) towards RHB, MB, and MO in mixture.

On the basis of the previous results, both LS and P-LS were tested for the removal of the three dyes in the mixture, stressing the conditions increasing the dye concentration in the solution from 10 ppm to 30 ppm. In order to reduce as much as possible the addition of chemicals in the matrix, all the tests were carried out at spontaneous pH (Figure 11).

As previously observed for the removal of single pollutants, at spontaneous pH, LS exhibited very high selectivity towards MB, reaching 99% of dye removal in 30 min, whereas, in these conditions, it maintained poor adsorption ability towards RHB and MO. In contrast, when employed in the removal of dyes in the mixture, P-LS did not retain its capability to indiscriminately remove cationic and anionic dyes. In fact, if, on the one hand, MO was still quantitatively removed after 30 min, the percentage of MB abatement was reduced to 75%, whereas only 34% of RHB was adsorbed after 150 min. These results can be reasonably attributed to a fast saturation of PANI and loofah sites by MO and MB, reducing, as a consequence, its activity towards RHB.

However, all these features can be profitably exploited to realize an innovative two-step approach for the selective separation and recovery of cationic and anionic dyes, as described below.

#### 3.2.4. Two-Step Adsorption Process for the Selective Removal of Dyes Mixture

The possibility to selectively recover different classes of substances from complex mixtures represents an important and innovative issue for waste valorization. In fact, if, on the one hand, the degradation of organic pollutants or their unselective adsorption from wastewater is an extensively investigated approach to reuse water for different applications, on the other hand, from a circular economy perspective, industrial wastewater should be considered as a potential source of useful chemicals. In this regard, the results obtained by LS and P-LS open promising perspectives.

**Figure 12:**
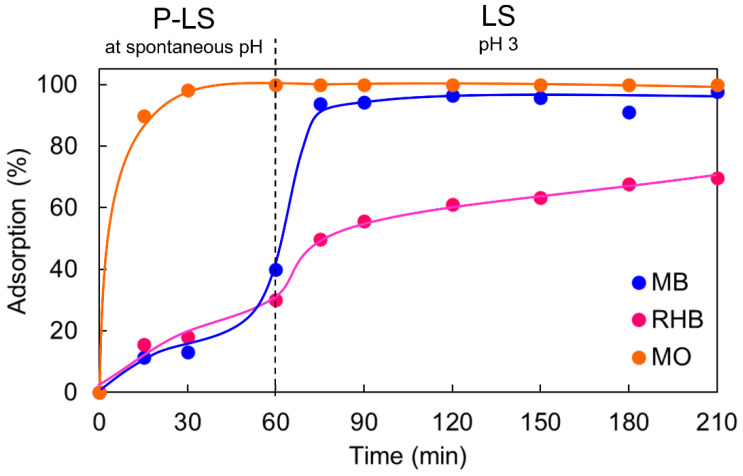
Selective adsorption of MO, MB, and RHB in mixture by a two-step pH-driven approach using P-LS and LS.

As reported above, the adsorption efficiency of P-LS towards MO is very high and fast, even when it is mixed with other dyes. This property can be effectively exploited. In fact, the material is able to adsorb, within 30 min, almost 100% of the anionic dye in solution (Figure 11b), also in the presence of other compounds. On the other hand, in acidic conditions (pH 3), LS shows the highest capability towards cationic dyes adsorption (Figure 6a,b). On the basis of these results, it is possible to design a two-step process consisting of: (i) the fast, selective removal of MO by P-LS at spontaneous pH, followed by (ii) the selective removal of cationic dyes carried out by replacing P-LS with LS and correcting the pH at 3 by properly adding HCl. Figure 12 displays the obtained results.

As shown in Figure 12, during the first 30 min, P-LS leads to the complete removal of MO from the solution, whereas the amount of cationic dyes removal is below 20%. However, the subsequent replacement of the adsorbent with LS and the lowering of the pH promotes the quantitative recovery of the cation components (100% MB and 70% RHB).

On the basis of our knowledge, this represents the first attempt at the selective removal of dyes in the mixture and paves the way for a new approach to wastewater treatment, focused no longer on compound degradation but on their recovery and sustainable valorization.

#### 3.2.5. Recycle Tests and Materials’ Regeneration

The possibility of reusing adsorbent materials is a hot topic for reducing waste production and process costs.

**Figure 13:**
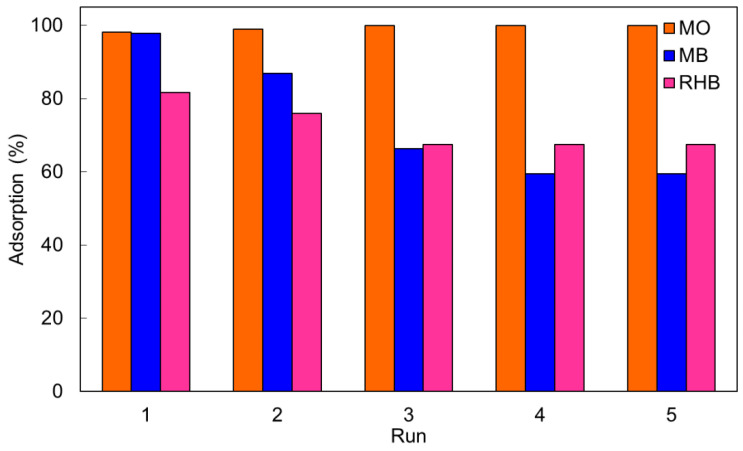
Adsorption by P-LS and LS under 5-run recycling.

To investigate the reusabilty of both LS and P-LS, these materials were tested with four recycling tests. In particular, as reported in the Expermental Section, each test consisted in using P-LS for the first 60 min, and then P-LS was removed from the solution and LS was added for another 2.5 h. As reported above, after each test both the adsorbents were washed with water, dried at room temperature, and reused for the subsequent run (Figure 13).

As it is possible to observe in Figure 13, P-LS maintains a very high level of MO adsorption for all the cycles, thus emerging as a valid alternative to traditional sorbents for the removal of this class of compounds. In contrast, after the first two cycles, LS gradually reduced its capability to adsorb cationic species, reasonably attributed to a gradual saturation of the active sites, even if, after 4 runs, its removal capability remained greater than 60%.

At the end of the last cycle, to release the adsorbed species and recover the original adsorption activity, both P-LS and LS underwent proper post-treatment based on two subsequent washings by both ammonia and HCl solutions, the amount of released dyes was properly evaluated, and the sorbent was reused (run 6).

**Figure 14:**
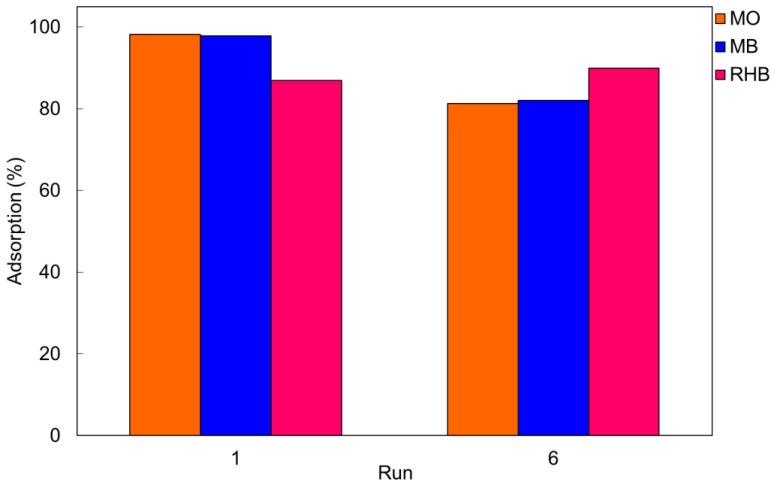
Comparison between the results of dyes adsorption using fresh materials (run 1) and after their regeneration (run 6).

As displayed in Figure 14, after the post-treatment, P-LS maintained high adsorption properties towards MO (100% and 81% during runs 1 and 6, respectively), as did LS towards both RHB (87% and 90% during runs 1 and 6, respectively) and MB (98% and 82% during runs 1 and 6, respectively).

Surprisingly, in contrast with the results reported in the scientific literature for PANI in powder form [30,31,32,33,56], P-LS did not release MO either by acidic washing or by an alkaline one.

This unusual behaviour suggests a complex interaction of the dye not only with the protonated amino groups of PANI through the sulphonic group but evidently also with loofah through the positive charge localized on the amino group, making hard its release. On the contrary, the two subsequent washings of LS led to the selective release of 50% of the adsorbed MB only under acidic conditions and the release of 50% of the adsorbed RHB only under alkaline conditions.

## 4. Conclusions

In this work, a polyaniline-coated loofah sponge (P-LS) was fabricated by in situ polymerization of aniline and properly characterized. The adsorption capability of both LS and P-LS was investigated for the removal of both anionic (methyl orange) and cationic (rhodamine and methylene blue) dyes, as single pollutant or a multi-dyes mixture, from aqueous solutions at different pH conditions.

It was demonstrated that, by modulating the adsorption pH, LS selectively adsorbs the cationic species in acidic conditions, maximizing the interactions between the surface charges of the sorbent and functional groups of the dyes, whereas it fails in the anionic dyes’ abatement. In contrast, thanks to its multifunctional characteristics, P-LS is capable of non-selectively removing all the investigated dyes from single-pollutant solutions at spontaneous pH. However, when used for the treatment of multi-dyes solution, P-LS exhibited a faster and more effective adsorbent capability towards the anionic species that was exploited for the development of a two-step adsorption process for the selective removal of dyes mixture. By this approach, during the first hour, P-LS was able to selectively remove 100% of the MO. The subsequent treatment by LS led to the abatement of 100% methylene blue and 70% rhodamine-B. The combined adsorbents system maintained high levels of dyes removal and good selectivity for multiple consecutive adsorption tests (five in total). The simple regeneration of the adsorbents and the concomitant controlled release of the adsorbed dyes led to the recovery of the initial sorption capability of the materials (81% for MO and *ca.* 85% for both RHB and MB, respectively) and, at the same time, the selective release of most of the adsorbed cationic dyes (50% of both the adsorbed MB and RHB), although the procedure failed regarding the release of the anionic component.

In conclusion, in the field of pollutants’ adsorption from water matrices, several highly performing materials have been proposed. However, they are generally based on activated carbons or other compounds usually used as dispersed powders. Here, for the first time, an easy, cheap, and fast approach for the selective adsorption/desorption of multi-dyes from aqueous solution was developed using innovative adsorbents based on a sustainable, abundant, and not-expensive material.

The interesting results should encourage the scientific community to investigate alternative strategies for wastewater treatment in line with a circular economy perspective.

## Figures and Tables

**Table 1:** Comparison among the results obtained in the present work and others from the scientific literature for the adsorption of different dyes on several loofah-based adsorbents.

Adsorbent	Dye	Q_max_ (mg·g^−1^)	Reference
PANI	Methyl orange	154.56	[58]
PANI-loofah cylindrica	Methyl orange	40.2	Present work
Crosslinked PANI	Methylene blue	13.85	[59]
Loofah cylindrica		47.00	[60]
Loofah cylindrica		1.5	Present work
PANI-loofah cylindrica		34.8	Present work
Loofah sponge	Trypan blue	114.94	[42]
ZnO-modified loofah sponge		129.87	
Loofah cylindrica	Reactive orange	50.00	[61]
Loofah cylindrica	Congo red	17.39	[16]
Loofah cylindrica	Malachite green	29.39	[39]
Loofah acutangula peel		50	[62]
Loofah aegyptica peel		166.67	[40]
NaOH-treated loofah aegyptica peel		161.29	

## Data Availability

The data that support the plots within this paper are available from the corresponding author on reasonable request.

## Supplementary Materials

The following supporting information can be downloaded at: https://www.mdpi.com/article/10.3390/polym14224897/s1, Figure S1: N_2_ adsorption/desorption isotherms at −196 °C of LS; Figure S2: Kinetic models for adsorption of dyes by LS and P-LS. RHB [(a) pseudo-first order (c) Elovich, (e) intraparticle diffusion (LS at pH 3); (b) pseudo-first order, (d) Elovich, (f) intraparticle diffusion (P-LS at spontaneous pH)], MO [(g) pseudo-first order, (h) Elovich, (i) intraparticle diffusion (P-LS at spontaneous pH)] and MB [(l) pseudo-first order m) Elovich, (n) intraparticle diffusion (LS at pH 3); (o) pseudo-first order, (p) Elovich, (q) intraparticle diffusion (P-LS at spontaneous pH)].
