# Supplementary material for: pH-Driven Selective Adsorption of Multi-Dyes Solutions by Loofah Sponge and Polyaniline-Modified Loofah Sponge
Source: Polymers (Basel). 2022 Nov 13;14(22):4897. doi: 10.3390/polym14224897 (PMC9693159; doi:10.3390/polym14224897)
Supplement: Supplementary file 1 [file polymers-14-04897-s001.zip › polymers-2000638-supplementary.pdf]

# Supporting Information

# pH-driven selective adsorption of multi-dyes solutions by loofah sponge and polyaniline-modified loofah sponge

**Melissa G. Galloni[1,2], Veronica Bortolotto[1], Ermelinda Falletta[1,2]*, Claudia L. Bianchi[1,2]**

1 Department of Chemistry, University of Milan, Via Golgi 19, 20133 Milan, Italy
2 Consorzio Interuniversitario Nazionale per la Scienza e Tecnologia dei Materiali (INSTM), Via Giusti 9, 50121, Florence, Italy
* Correspondence: ermelinda.falletta@unimi.it; Tel.: +39-02-503114410

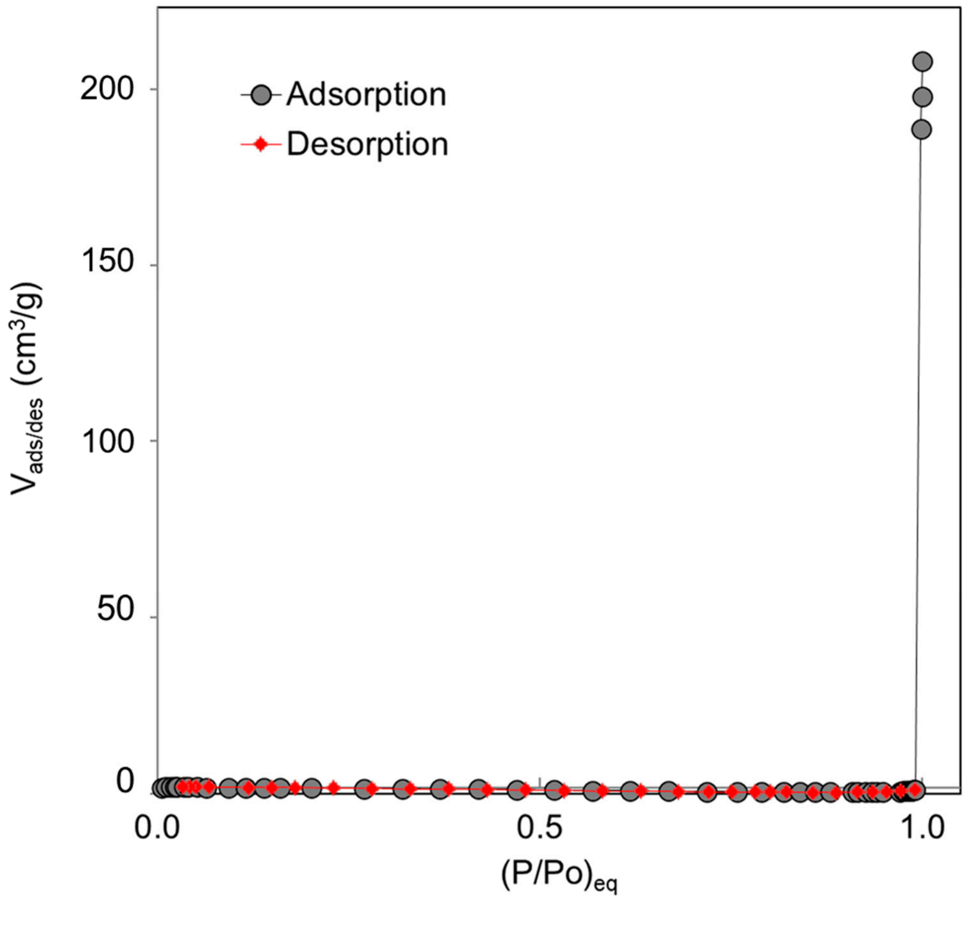


**Figure S1.** $N_2$ adsorption/desorption isotherms at -196°C of LS.

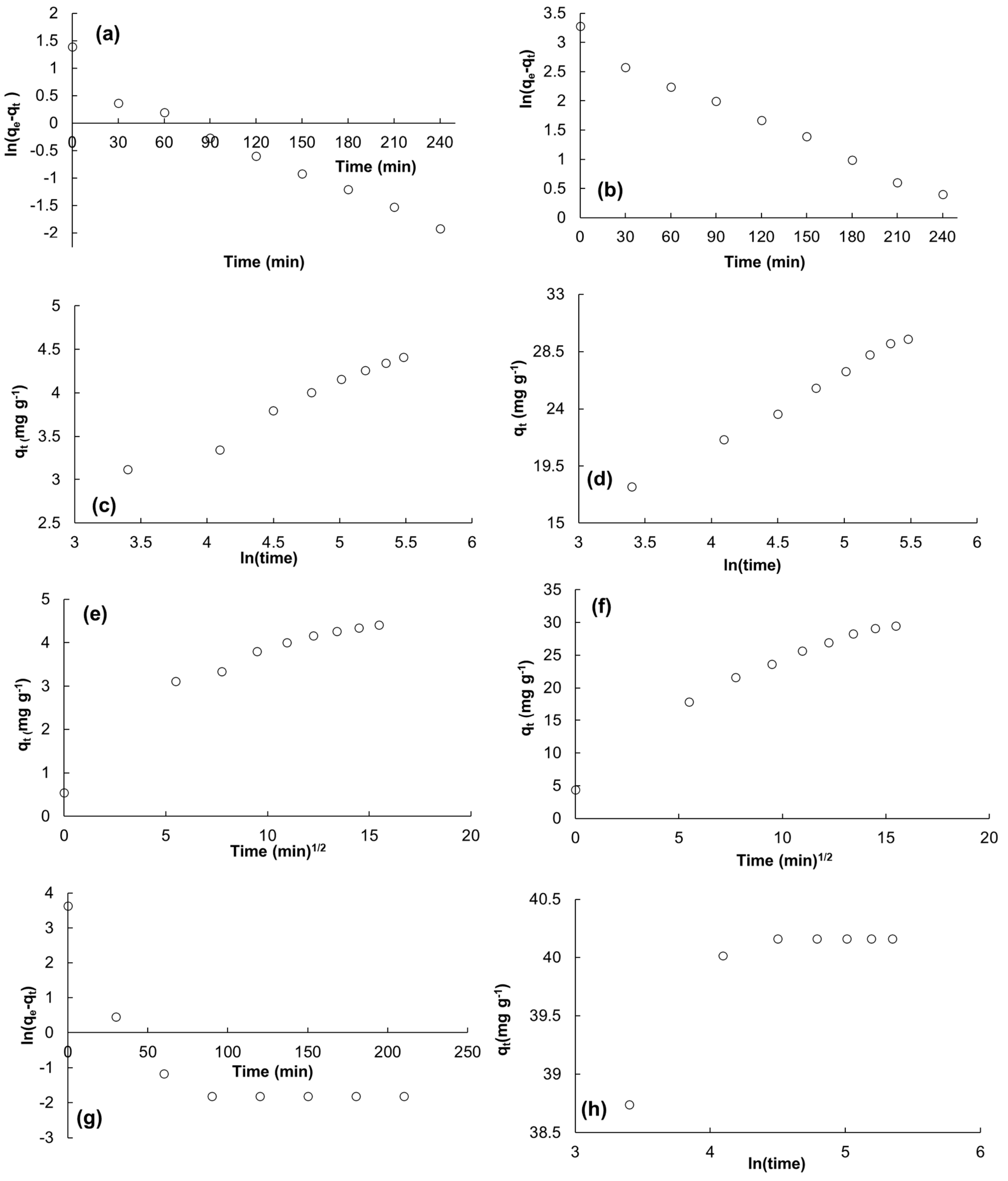
(a)
ln(qe-qt )
Time (min)
(b)
ln(qe-qt)
Time (min)
(c)
qt (mg g-1)
ln(time)
(d)
qt (mg g-1)
ln(time)
(e)
qt (mg g-1)
Time (min)1/2
(f)
qt (mg g-1)
Time (min)1/2
(g)
ln(qe-qt)
Time (min)
(h)
qt(mg g-1)
ln(time)

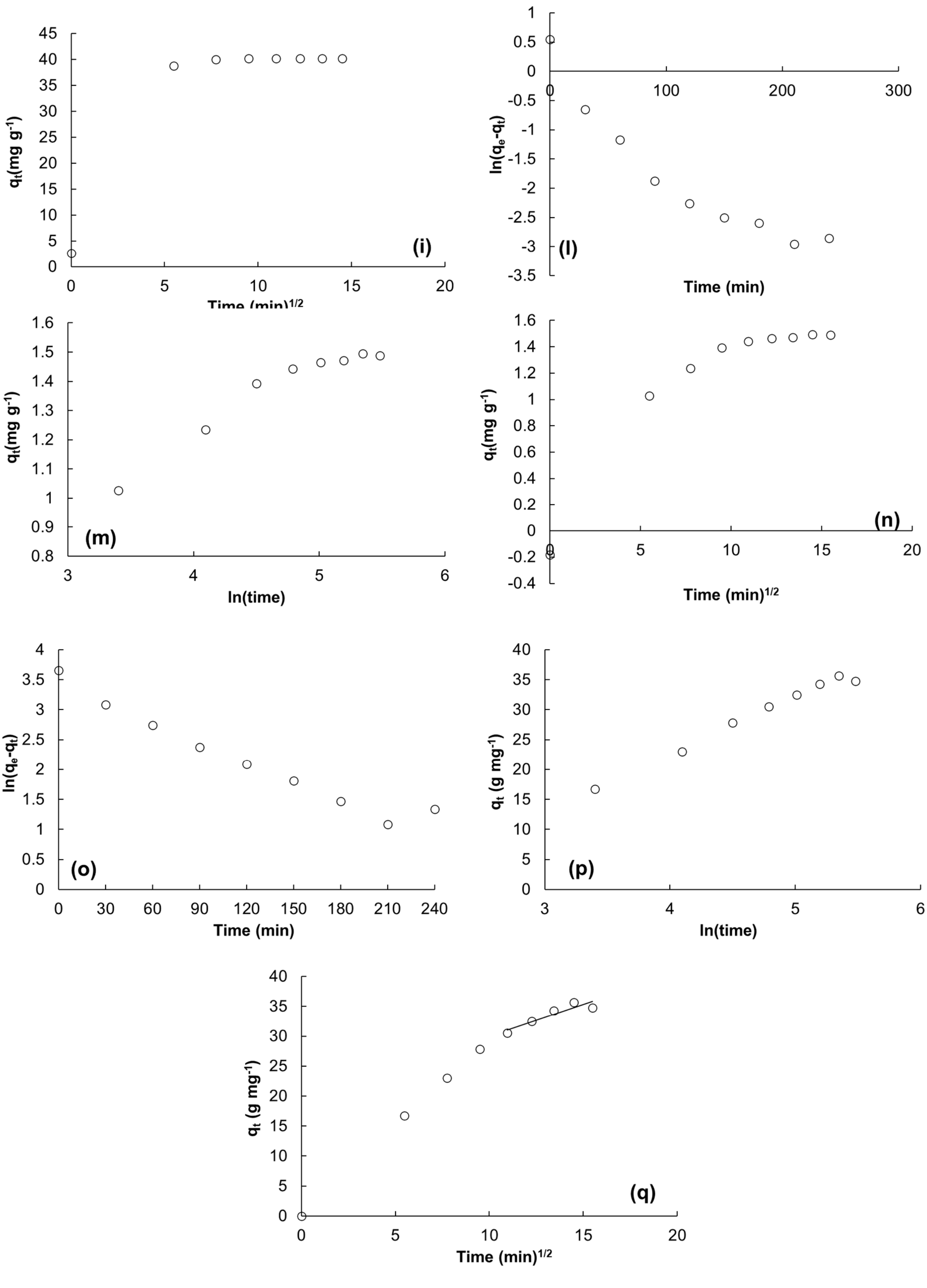


**Figure S2:** Kinetic models for adsorption of dyes by LS and P-LS. RHB [a) pseudo-first order c) Elovich, e) intraparticle diffusion (LS at pH 3); b) pseudo-first order, d) Elovich, f) intraparticle diffusion (P-LS at spontaneous pH)], MO [g) pseudo-first order, h) Elovich, i) intraparticle diffusion (P-LS at spontaneous pH)] and MB [l) pseudo-first order m) Elovich, n) intraparticle diffusion (LS at pH 3); o) pseudo-first order, p) Elovich, q) intraparticle diffusion (P-LS at spontaneous pH)].
